# Relationship between vertical facial patterns and palatal morphology in class I and class II malocclusion - An in vitro study

**DOI:** 10.6026/973206300211734

**Published:** 2025-05-31

**Authors:** Abhilasha Patidar, Harshavardhan Reddy, Shekhar K. Asarsa, Mansi Mehta, Drashti Chikhalia, Richa Singh

**Affiliations:** 1 Department of Orthodontics and Dentofacial Orthopedics, College of Dental Science and Hospital, Indore, Madhya Pradesh, India; 2 Department of Orthodontics and Dentofacial Orthopedics, HKE's S. Nijalingappa Dental College, Kalaburagi, Karnataka - 585105, India; 3 Department of Orthodontics and Dentofacial Orthopedics, Siddhpur Dental College and Hospital, Dethali, Patan, Gujarat, India; 4 Department of Orthodontics and Dentofacial Orthopedics, Sai Dental Clinic, Abrama, Valsad, Gujarat, India; 5 Department of Orthodontic and Dentofacial Orthopedics, Chandra Dental College and Hospital, Safedabad, Barabanki Uttar Pradesh, India

**Keywords:** Vertical facial growth patterns, palatal morphology, class I malocclusion, class II malocclusion, mandibular plane angle (MPA), facial height (FH), palatal width (PW1), palatal arch length (PAL), orthodontic treatment planning, maxillary arch development

## Abstract

The relationship between vertical facial growth patterns and palatal morphology in Class I and Class II malocclusion cases is explored. An in vitro analysis of 24 subjects (12 in each group) showed that individuals with Class II malocclusion and high-angle growth displayed significantly greater mandibular plane angles, facial heights, palatal widths, and arch lengths compared to their Class I counterparts. Data shows that vertical growth tendencies have a marked influence on palatal dimensions. Visual data representation further confirmed the distinctive morphological differences between the groups. Thus, the need to account for vertical facial growth patterns in orthodontic diagnostics and treatment planning is reported.

## Background:

The relationship between vertical facial patterns and palatal morphology has been a subject of growing interest in orthodontics, regarding malocclusions such as Class I and Class II [1, 2]. Class I malocclusion typically presents with a balanced facial growth pattern, where the upper and lower jaws align correctly and any misalignment exists within the teeth themselves. This type of malocclusion is usually associated with a low-angle or horizontal vertical facial pattern, where the facial height is relatively proportional [3]. On the other hand, Class II malocclusion is characterized by a skeletal imbalance, where the upper jaw or teeth protrude significantly over the lower jaw, often leading to an open bite and increased lower facial height [4]. Class II patients typically exhibit a high-angle vertical growth pattern, meaning they have a steeper mandibular plane and a longer lower facial height. This difference in vertical facial patterns has a direct influence on the development of the palate. Palatal morphology, which includes aspects like palatal width and arch length, plays a critical role in determining overall dental function and alignment [5, 6]. Variations in vertical facial growth can lead to differences in the maxillary arch's structure, with high-angle individuals tending to have wider palates and longer arches, while low-angle individuals may exhibit narrower, more compact palates [7].

Understanding these relationships is essential for orthodontists, as vertical growth patterns significantly affect the types of orthodontic treatments that might be most effective for patients. For instance, Class II patients with high-angle growth patterns may require interventions that account for their increased vertical dimension, possibly involving maxillary expansion or the use of growth-modifying appliances [8]. Conversely, Class I patients with low-angle growth patterns may need treatment plans that focus on expanding the arch or correcting any occlusal issues while maintaining a stable vertical relationship [9]. Therefore, it is of interest to investigate how vertical facial growth patterns influence palatal morphology by comparing Class I and Class II malocclusion cases.

## Materials and Methods:

This in-vitro study included a total of 24 samples, equally divided into 12 Class I and 12 Class II malocclusion cases. The primary objective was to evaluate the relationship between vertical facial patterns and palatal morphology. The samples were carefully selected based on the following criteria: participants were aged between 12 and 18 years, with an exclusion of patients who had craniofacial syndromes, missing teeth, or a history of previous orthodontic treatment, ensuring the homogeneity of the sample. Data collection focused on two key areas: vertical facial patterns and palatal morphology. For vertical facial patterns, measurements such as the mandibular plane angle (MPA) and facial height (FH) were recorded, alongside other relevant indicators that reflect the vertical growth pattern. In terms of palatal morphology, the study assessed palatal width at the first molar (PW1) and palatal arch length (PAL) to determine how these features correlated with the underlying vertical facial structure. These measurements were essential in analyzing the palatal development and how it may vary between Class I and Class II malocclusion groups, providing valuable insights into the impact of vertical growth patterns on the maxillary arch.

## Results:

The demographic characteristics of the study sample, which consisted of 24 participants (12 Class I and 12 Class II malocclusion cases), were analyzed. The average age of the Class I group was 14.2 ± 1.3 years, while the Class II group had a mean age of 14.5 ± 1.4 years, showing no significant age differences between the two groups. Gender distribution was relatively balanced in both groups, with the Class I group consisting of 6 males and 6 females and the Class II group having 5 males and 7 females. When assessing the vertical facial patterns, mandibular plane angle (MPA) was significantly higher in the Class II group (32.1 ± 4.5°C) compared to the Class I group (26.4 ± 3.2°C), indicating a steeper growth pattern associated with the high-angle vertical growth seen in Class II malocclusion. Additionally, facial height measurements revealed that the Class II group had a greater average facial height (14.3 ± 2.0 cm) compared to the Class I group (12.5 ± 1.8 cm), further supporting the association between increased lower facial height and Class II malocclusion (Table 1). The palatal morphology measurements were taken for both groups, specifically assessing palatal width at the molar (PW1) and palatal arch length (PAL). For palatal width, the Class II group exhibited a larger average width of 48.3 ± 3.0 mm compared to the Class I group, which had an average width of 45.2 ± 2.1 mm. This finding aligns with the assumption that patients with higher mandibular plane angles and vertical growth patterns tend to have wider palates. Similarly, the palatal arch length was greater in the Class II group (39.5 ± 2.0 mm) compared to the Class I group (36.8 ± 1.5 mm), suggesting that the increased vertical facial height in Class II cases might contribute to a longer maxillary arch (Table 2). The water plot graph provides a visual comparison between palatal width (PW1) and palatal arch length (PAL) for both Class I and Class II malocclusion cases, based on their vertical facial patterns. The x-axis of the graph represents palatal width (measured at the first molar), while the y-axis represents palatal arch length. In the graph, the blue dots represent the Class I cases and the red dots correspond to the Class II cases. From the graph, it is apparent that Class II malocclusion cases, which are associated with a high-angle vertical facial pattern, tend to exhibit wider palates (higher values on the x-axis) and longer palatal arches (higher values on the y-axis) compared to Class I malocclusion cases, which are linked with a more horizontal facial growth pattern. The color coding further distinguishes the relationship between vertical growth patterns and palatal morphology, highlighting the trend of increased palatal width and arch length in the high-angle Class II cases. Overall, the study demonstrates that vertical facial patterns have a significant influence on palatal morphology, with Class II patients (high-angle pattern) showing wider and longer palates compared to Class I patients (low-angle pattern). These findings provide important insights for orthodontic treatment planning, particularly in determining how malocclusions and facial growth patterns influence the development of the maxillary arch (Figure 1 - see PDF). The water plot reveals a discernible pattern wherein individuals with Class II malocclusion, typically associated with a high-angle vertical growth trajectory, exhibit notably broader and more elongated palatal arches in comparison to their Class I counterparts with low-angle growth patterns. This graphical representation underscores the morphological interplay between vertical facial growth dynamics and palatal architecture.

## Discussion:

Malocclusions, particularly Class I and Class II, are commonly encountered in orthodontic practice. They are characterized by misalignments of the teeth and jaws, affecting both aesthetics and function [10]. The vertical growth patterns of the face play a pivotal role in determining the overall shape and size of the palate. Class I malocclusions generally exhibit a more balanced vertical growth pattern, whereas Class II malocclusions are often associated with a high-angle vertical pattern, leading to a more pronounced vertical dimension of the face [11]. Vertical facial patterns refer to the orientation and relationship of the upper and lower jaws in the vertical dimension. In Class II malocclusions, the mandibular plane angle (MPA) is typically larger, indicating increased vertical growth. Conversely, Class I malocclusions usually display a more horizontal growth pattern, where the mandibular plane angle is smaller, indicating a more stable and balanced facial height [12]. The palatal morphology, specifically palatal width and arch length, has significant implications in orthodontic treatment planning. A broader and longer palate is often observed in individuals with high-angle vertical growth, such as those with Class II malocclusions. This increased palatal width may be a compensatory adaptation to the vertical excess in the lower face and it can be a factor influencing orthodontic treatment approaches, such as the need for maxillary expansion or arch development [13]. On the other hand, Class I malocclusion patients, exhibiting a balanced growth pattern, typically have more compact palates with less pronounced vertical growth, requiring less intensive interventions to achieve functional and aesthetic alignment [14]. Understanding the relationship between vertical facial patterns and palatal morphology is crucial in designing effective and personalized orthodontic treatment strategies. The vertical dimension affects not only the aesthetic appearance of the face but also the overall health of the oral cavity, influencing occlusal relationships, airway function and even speech. This study aims to explore how vertical facial patterns impact the shape and size of the palate, focusing on Class I and Class II malocclusions. By evaluating key variables such as mandibular plane angle (MPA), facial height (FH), palatal width and arch length, this research seeks to shed light on the underlying anatomical differences and their implications for orthodontic treatment. The results of this study emphasize the significant relationship between vertical facial patterns and palatal morphology in Class I and Class II malocclusions. Class II malocclusions, often characterized by a high-angle vertical facial pattern and were found to have significantly wider palates and longer palatal arches than Class I malocclusions, which typically exhibit a more balanced or horizontal facial growth pattern. These findings align with previous research indicating that increased vertical growth, such as that seen in Class II patients, leads to greater palatal expansion. The higher mandibular plane angle and increased facial height observed in Class II malocclusions likely contribute to the development of these wider and longer palates, which may require distinct orthodontic treatment approaches, especially concerning maxillary expansion or arch development.

Eckmüller *et al.* supports the notion that Class II malocclusions, with their high-angle patterns, tend to show more vertical growth and broader arches when compared to Class I malocclusions, which typically have a horizontal growth pattern [15]. The increased arch width and length in Class II patients may be associated with compensatory mechanisms aimed at maintaining facial harmony despite excessive vertical growth. Therefore, clinicians must consider these differences in palatal morphology when planning orthodontic interventions. In contrast, the results for Class I malocclusion patients, who displayed relatively smaller palatal widths and shorter arch lengths, suggest that these patients, having a more balanced vertical growth pattern, may require orthodontic treatment strategies focused on maintaining the stability of the existing palatal structure while achieving functional alignment. These findings are consistent with research by Adhikari *et al.* (2020), who indicated that Class I cases are generally more stable in terms of palatal morphology due to the balanced growth patterns observed in such individuals [16]. Overall, the findings from this study suggest that vertical facial patterns significantly influence palatal morphology and orthodontists must tailor their treatment strategies based on these structural differences. Specifically, for Class II malocclusions, treatment plans should account for the potential need for maxillary expansion and arch lengthening, whereas Class I patients may benefit from more subtle adjustments to maintain arch integrity while achieving occlusal harmony. Further literature supports these conclusions, suggesting that palatal expansion is more pronounced in Class II patients with increased mandibular plane angles and the malocclusion's impact on facial aesthetics and function should be addressed comprehensively in the treatment planning phase (Volk *et al.* 2010; Achmad *et al.* 2022) [17, 18]. There is a significant correlation between palatal width and vertical facial patterns. Hypo-divergent individuals tend to have greater intermolar widths, whereas hyper-divergent individuals exhibit narrower palates. Class II subjects demonstrate greater posterior palatal height compared to Class I subjects. Conversely, Class I individuals have a significantly larger palatal surface area. No notable differences in palatal volume were observed across different malocclusion classes or vertical facial patterns [19].

## Conclusion:

The significant relationship between vertical facial patterns and palatal morphology in Class I and Class II malocclusions is shown. Understanding these relationships is crucial for orthodontists in tailoring treatment approaches, particularly when dealing with arch development and expansion in growing patients. Further studies with larger sample sizes and longer follow-up periods are necessary to confirm these findings and refine orthodontic treatment protocols.

## Figures and Tables

**Table 1:** Demographics of patients

**Demographic Characteristic**	**Class I (n=12)**	**Class II (n=12)**
Age (mean ± SD)	14.2 ± 1.3 years	14.5 ± 1.4 years
Gender (Male/Female)	6-Jun	7-May
Mandibular Plane Angle (°)	26.4 ± 3.2	32.1 ± 4.5
Facial Height (cm)	12.5 ± 1.8	14.3 ± 2.0

**Table 2:** Treatment arm

**Treatment Parameter**	**Class I (n=12)**	**Class II (n=12)**
Palatal Width at Molar (mm)	45.2 ± 2.1	48.3 ± 3.0
Palatal Arch Length (mm)	36.8 ± 1.5	39.5 ± 2.0
